# In Vivo Microscopic Features of Acne vulgaris Revealed via LC‐OCT

**DOI:** 10.1111/srt.70371

**Published:** 2026-06-15

**Authors:** Daniele Omar Traini, Gerardo Palmisano, Alessandro Di Stefani, Enrico Bocchino, Cristina Guerriero, Mariano Suppa, Ketty Peris

**Affiliations:** ^1^ Department of Dermatology Agostino Gemelli University Hospital Rome Italy; ^2^ Department of Translational Medicine and Surgery Catholic University of The Sacred Heart Rome Italy; ^3^ Department of Dermatology Hôpital Erasme, HUB, Université Libre de Bruxelles Brussels Belgium; ^4^ Department of Dermatology, Institut Jules Bordet, HUB Université Libre de Bruxelles, HUB Brussels Belgium; ^5^ Groupe d'imagerie cutanée noninvasive (GICNI) Société Française de Dermatologie (SFD) Paris France

**Keywords:** acne, imaging, LC‐OCT, Line field confocal optical coherence tomography

## Abstract

**Background:**

Acne vulgaris is a common skin disorder characterized by multifactorial pathogenesis. In recent years, multiple non‐invasive imaging methods have been explored to monitor in vivo acne lesions. Technologies such as reflectance confocal microscopy (RCM) and optical coherence tomography (OCT) have improved our capabilities to characterize and monitor this condition; nevertheless, limitations remain in resolution and depth. Line‐field confocal optical coherence tomography (LC‐OCT) is a novel imaging technique that combines the strengths of RCM and OCT, offering high‐resolution vertical and horizontal imaging of skin tissue up to 500 µm deep. The aim of this study was to characterize and quantify the microstructural features of different acne lesion morphologies using LC‐OCT and to compare these findings with those observed in normal skin.

**Methods:**

A total of 52 facial acne lesions (microcomedones, closed and open comedones, papules, and pustules) from 20 patients with mild‐to‐moderate acne and 15 healthy controls were imaged using LC‐OCT. Vertical, horizontal, and 3D images were analyzed for structural features, with quantitative measurements of lesion diameter and depth.

**Results:**

LC‐OCT revealed distinct microstructural features at each stage of acne development. Microcomedones showed bright perifollicular rings in 83.3% of cases, absent in healthy skin, indicating early follicular hyper keratinization. Closed comedones appeared as cystic, keratin‐filled structures beneath intact epidermis, while open comedones displayed surface discontinuities and mixed reflectivity plugs. Papules and pustules demonstrated perifollicular hyperreflective halos and numerous “bright dots”, corresponding to inflammatory cell infiltrates.

**Conclusions:**

LC‐OCT provides non‐invasive, high‐resolution imaging of acne microarchitecture, from subclinical changes in acne‐prone skin and incipient microcomedones to inflammatory lesions, offering potential utility in diagnosis, disease monitoring and personalized therapeutic strategies.

## 1 Introduction

Acne vulgaris is a common inflammatory skin disease clinically characterized by comedones, papules, pustules and nodules. The initial pathogenetic mechanism consists of an abnormal follicular keratinization and retention of keratin within the follicle, which lead to the formation of microcomedo [1]. As sebum production increases and *Cutibacterium acnes* (also known as *Propionibacterium acnes*) proliferates within the follicle, the microcomedo can evolve into a visible closed or open comedo, with continuous accumulation of keratin and enlargement of the follicular duct [2, 3].

In the last decade, non‐invasive skin imaging techniques as reflectance confocal microscopy (RCM) and optical coherence tomography (OCT) have increasingly been used to investigate acne pathophysiology in vivo [2, 4]. RCM enables horizontal visualization of the epidermis and upper dermis at near‐cellular resolution (0.5–1 µm), allowing observation of enlarged follicles in comedones as well as inflammatory cell infiltrates in papules and pustules. RCM also displays characteristic findings in clinically normal skin of acne patients; specifically, “bright rings” correspond to hyperkeratotic linings of follicles (incipient microcomedones) [5, 6, 7]. However, RCM imaging is typically horizontal (en face) and limited to 200 µm depth, which may not fully capture deeper dermal changes or the 3D architecture of follicles [8]. Conventional OCT, on the other hand, provides vertical images up to 1 mm deep, which can depict the overall architecture of comedones and papules but with lower resolution (7–15 µm). Studies focused on acne using OCT have reported distinctive patterns such as the “reverse V‐shaped” appearance of closed comedones in vertical section and a “rectangular” superficial hyperreflective plug in open comedones, [6, 9] as well as differences in dermal reflectivity or vascular signal between non‐inflamed and inflamed lesions [6, 9]. However, the lower resolution represents a major limitation of standard OCT, preventing visualization of cellular structures and early alterations [9].

Line‐field confocal optical coherence tomography (LC‐OCT) is a recently introduced imaging technology that combines the advantages of RCM and OCT [10]. LC‐OCT can produce ultrahigh‐resolution vertical images, approaching RCM resolution, with an imaging depth of up to about 500 µm, as well as horizontal and three‐dimensional (3D) reconstructions of the skin tissue [11]. An integrated dermoscopic camera allows a precise correlation of the LC‐OCT image with clinical‐dermoscopic images. Thus, LC‐OCT can explore skin in vivo producing a “virtual biopsy” [12, 13].

We applied LC‐OCT for the first time to examine various acne lesions in vivo, including non‐inflammatory microcomedones, closed and open comedones, as well as inflammatory lesions, such as papules and pustules. The aim of this study was to characterize the in vivo LC‐OCT features of different acne lesion morphologies (microcomedones, open and closed comedones, papules and pustules), to quantify lesion dimensions, and to compare these findings with normal skin.

## 2 Materials and Methods

### 2.1 Study Design and Patients

Patients with untreated mild‐to‐moderate acne (comedonal and papulopustular lesions) were recruited from the outpatient clinic of the Dermatology Department, Fondazione Policlinico Universitario Agostino Gemelli IRCCS (Rome, Italy). We included patients of both sexes, presenting with mild‐to‐moderate acne, who were not receiving any current topical or systemic acne treatment. Nodular acne was excluded, as the depth of nodular lesions typically extends well into the deeper dermis and sometimes the subcutaneous tissue (>1–2 mm), exceeding the maximum penetration depth of LC‐OCT [14]. Additionally, the normal skin of 15 age‐matched healthy volunteers without any history of acne was evaluated by LC‐OCT for comparison. All imaging acquisitions were conducted on the face. A thin layer of paraffin oil was applied to the skin to optimize optical coupling. LC‐OCT (DeepLive, DAMAE Medical, France) was used to perform non‐invasive skin imaging. Images were analyzed by three independent investigators and any disagreement was resolved by consensus. Because the study was descriptive and image acquisition and interpretation were performed within the same research group, formal blinding to clinical information was not implemented. For each lesion and unaffected skin, we acquired LC‐OCT images in vertical, horizontal and three‐dimensional modes. LC‐OCT sections were evaluated with reference to the histopathological and RCM hallmarks for acne [5]. Additional LC‐OCT criteria were introduced when required.

Six qualitative morphological features visible on LC‐OCT images (bright perifollicular ring, epidermal discontinuity, hyperreflective bright dots, hyperreflective halo, fluid‐filled cavity, and pseudo membrane) were assessed for each lesion and recorded as present or absent. We also performed quantitative measurements using the device's software calipers: specifically, we measured the infundibulum diameter (maximum horizontal width of the follicular lumen or lesion cavity) and the lesion depth (vertical distance from skin surface to the deepest extent of visible lesion structure) for each image. For pustules, the diameter of the pus‐filled cavity was measured.

Continuous variables were expressed as mean ± standard deviation (SD). Quantitative measurements of lesion dimensions were compared using Student's *t*‐test. The presence or absence of LC‐OCT morphological features was treated as categorical data and compared across the six study groups (normal skin, microcomedones, closed comedones, open comedones, papules, and pustules) using Fisher's exact test. A *p*‐value < 0.05 was considered statistically significant. All patients provided written informed consent. Approval of this study was obtained from the Local Ethics Committee—Comitato Etico Territoriale Lazio Area 3 (Prot ID: 2983).

## 3 Results

We included 52 lesions of 20 patients (12 females, 8 males; mean age 21 ± 2 years) with untreated facial acne vulgaris, and 15 healthy volunteers (10 females and 5 males; mean age 21 years ± 2 years). In detail, acne lesions included 12 microcomedones, 12 closed and 10 open comedones, 8 papules and 10 pustules. Quantitative assessment showed that microcomedones had a mean diameter of 110 ± 30 µm and a depth of 80 ± 20 µm. Closed comedones measured 310 ± 60 µm in diameter and 180 ± 50 µm in depth, whereas open comedones averaged 420 ± 70 µm in diameter with a depth of 250 ± 60 µm. Papules exhibited an average lateral inflammatory zone of 600 ± 100 µm and a depth of 280 ± 60 µm. Pustules featured a pus‐filled cavity measuring 290 ± 50 µm in diameter and extending to a depth of 400 ± 80 µm.

**FIGURE 1:**
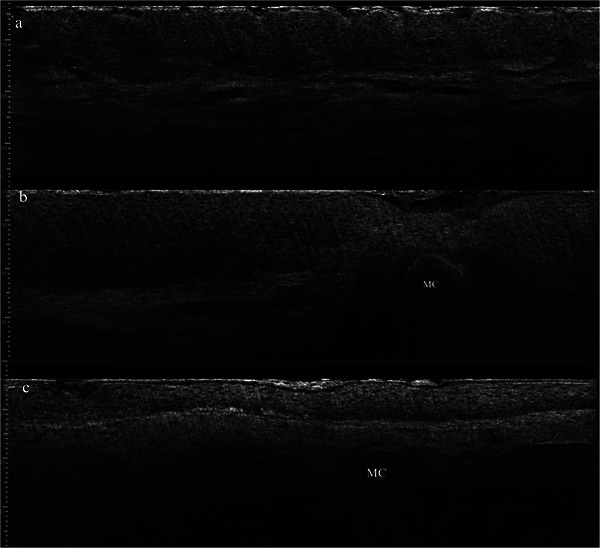
Healthy skin (1a) in comparison with acne‐prone skin where are visible (1b and 1c) microcomedones (MC).

**FIGURE 2:**
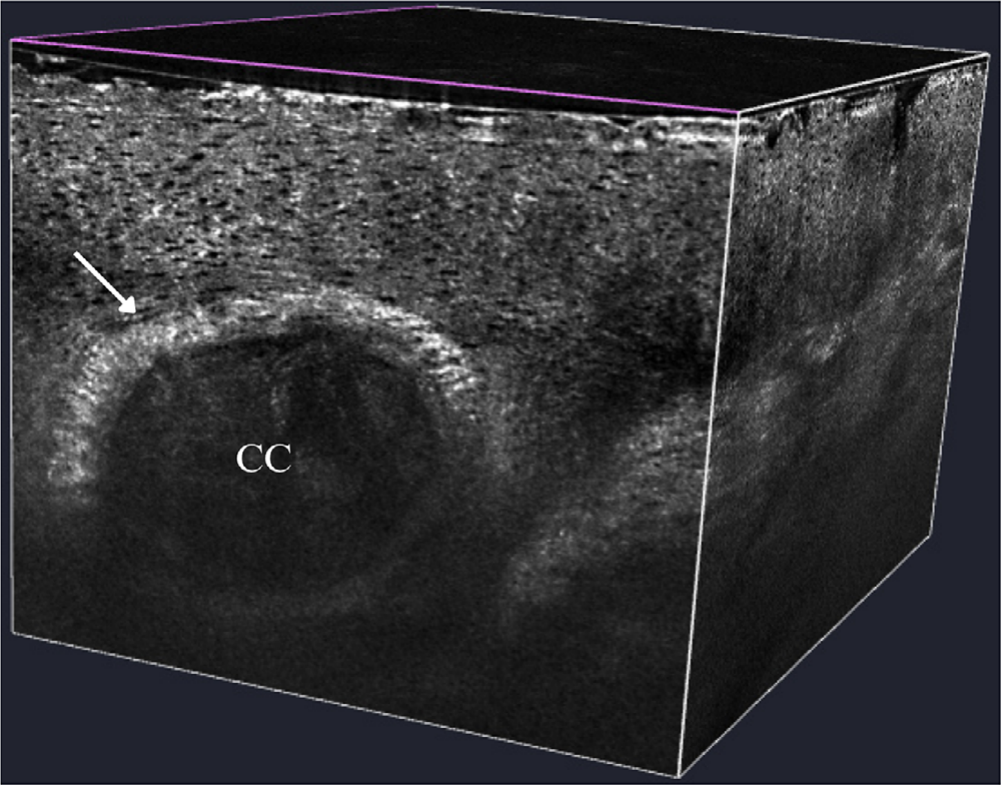
LC‐OCT depicted closed comedones (CC) as enclosed follicular cysts with reflective keratin walls (white arrow) and contents, an intact epidermal cover, and an absence of inflammatory changes.

**FIGURE 3:**
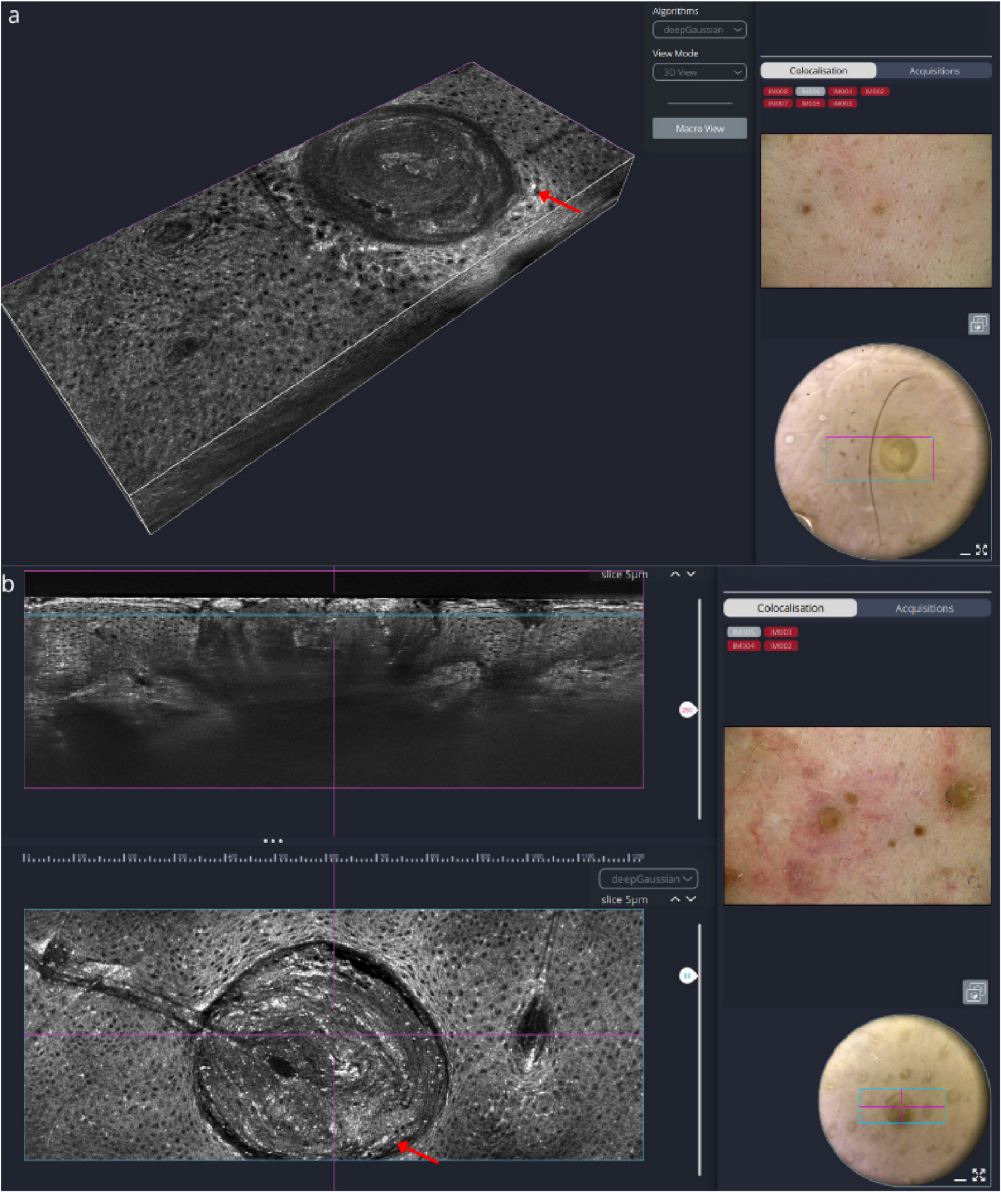
LC‐OCT 3D reconstructions (a), vertical (b, upper panel) and horizontal images(b, lower panel) of an open comedo revealed a lumen filled with abundant amorphous material of mixed reflectivity containing “bright dots” (red arrows), likely corresponding to microcalcifications and trapped inflammatory cells.

**FIGURE 4:**
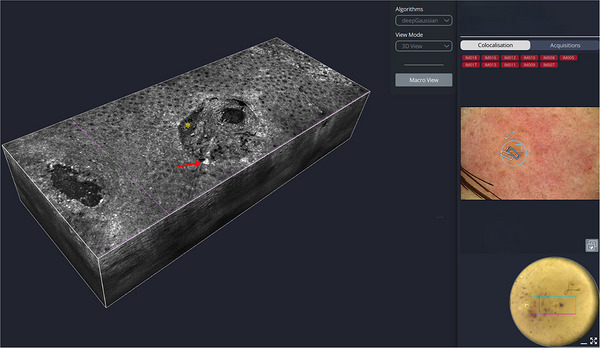
LC‐OCT of papules reveals numerous bright dots and globules scattered in and around the follicular area (red arrow). These highly reflective small particles (5–20 µm) may represent inflammatory cells (primarily neutrophils) and their aggregates. In papules, these cells were found within the follicular epithelium and lumen (exocytosis) as well as in the perifollicular dermis. Surrounding the follicular area, the dermis showed a cloudy, hyperreflective appearance, that could be consistent with a perifollicular inflammatory infiltrate and associated edema (white asterisk).

**FIGURE 5:**
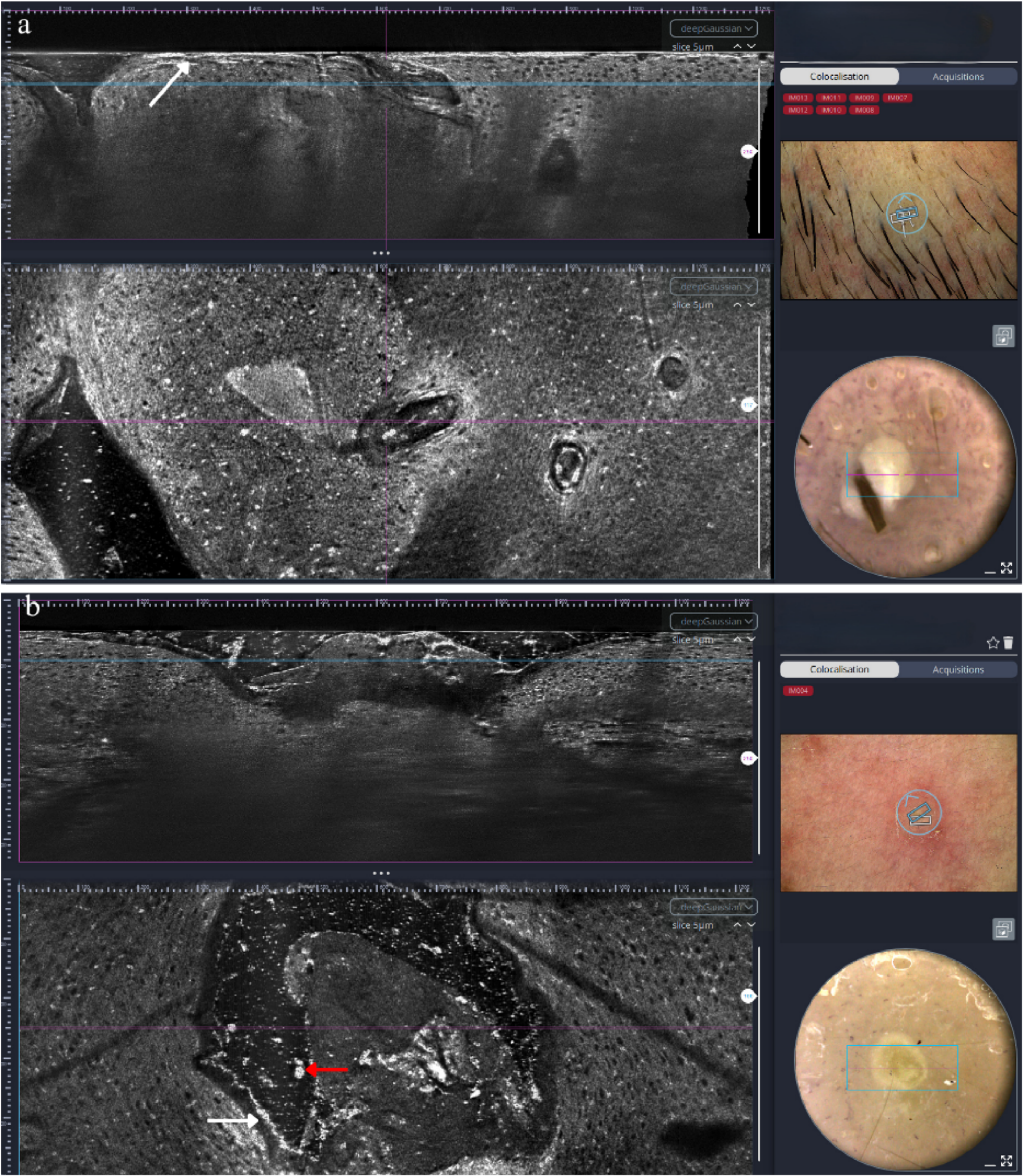
LC‐OCT of pustules (a,b) shows a superficial, round‐to‐oval low‐reflectivity cavity (200–400 µm in diameter) filled with numerous bright dots (dense inflammatory cells, red arrow), capped by a thin epidermal roof, and surrounded by a hyperreflective perifollicular inflammatory halo. A subtle layered reflectivity within the fluid pocket (brighter at the margins where leukocytes concentrate and darker centrally where fluid predominates) creates a “pseudo‐membrane” (white arrows).

A bright perifollicular ring was observed in 83.3% of microcomedones (10/12; *p* < 0.05) and 92% of open comedones (11/12; *p* = 0.06), but it was absent in normal skin Figure 1. Epidermal surface discontinuity was most present in open comedones (100%; 12/12; *p* < 0.05), less frequent in pustules (30%; 3/10) and papules (25%; 2/8), yet undetectable in closed comedones Figures 2 and 3. Hyperreflective bright dots and hyperreflective halos surrounding the lesions were associated with 8/8 papules (100%; *p* ≤ 0.05) and 10/10 pustules (100%; *p* < 0.05), but rare (≤ 20%) in non‐inflammatory lesions Figure 4. A fluid‐filled cavity was exclusively present in all 10 pustules (100%; *p* < 0.05), sometimes associated with a layered appearance and a white “pseudo‐membrane” at the border (7/10; 70%; *p* < 0.05) Figure 5. Key characteristics of the lesions are summarized in Table 1. In normal skin, all the features were absent: there was no evidence of follicular hyperkeratosis or bright perifollicular ring formation, nor any detectable signs of inflammation.

**TABLE 1:** Key features of acne under LC‐OCT and their prevalence across the different lesions.

	Normal skin	Microcomedones	Open comedones	Closed comedones	Papules	Pustules
Bright ring	0/15 (0%) (*p* = 0.0284)	10/12 (83,3%) (*p* = 0.0000)	11/12 (91.7) (*p* = 0.0578)	4/10 (40%) (*p* = 0.1983)	0/8 (0%) (*p* = 0.1887)	0/10 (0%) (*p* = 0.1057)
Bright dots	0/15 (0%) (*p* = 0.0005)	3/15 (0.2%) (*p* = 0.5120)	2/12 (16.7%) (*p* = 0.1861)	2/10 (20%) (*p* = 0.3000)	8/8 (100%) (*p* = 0.0002)	10/10 (100%) (*p* = 0.0000)
Hyperreflective halo	2/15 (13%) (*p* = 0.0646)	0/12 (0%) (*p* = 0.0029)	2/12 (16.7%) (*p* = 0.1875)	2/10 (20%) (*p* = 0.3108)	8/8 (100%) (*p* = 0.0001)	10/10 (100%) (*p* = 0.0000)
Fluid‐filled cavities	0/15 (0%) (*p* = 0.0586)	0/12 (0%) (*p* = 0.1919)	0/12 (0%) (*p* = 0.1919)	0/10 (0%) (*p* = 0.1946)	1/8 (12,5%) (*p* = 1.0000)	10/10 (100%) (*p* = 0.0000)
Hyperreflective Pseudomembrane	0/15 (0%) (*p* = 0.3351)	0/12 (0%) (*p* = 0.3360)	0/12 (0%) (*p* = 0.3360)	0/10 (0%) (*p* = 0.5827)	0/8 (0%) (*p* = 0.5855)	7/10 (70%) (*p* = 0.0000)
Epidermal discontinuity at the surface	0/15 (0%) (*p* = 0.0078)	0/12 (0%) *(p* = 0.0281)	12/12 (100%) (*p* = 0.0000)	0/10 (0%) (*p* = 0.0546)	2/8 (25%) (*p* = 1.0000)	3/10 (30%) (*p* = 0.7056)

## 4 Discussion

In acne patients, areas of skin that appeared clinically normal showed subtle microscopic changes on LC‐OCT consistent with microcomedones formation. We examined 20 sites of apparently normal skin in acne patients (one per patient) and compared them to 15 sites from healthy controls. LC‐OCT vertical sections of acne patients’ normal skin showed one or more follicular units with slight infundibular dilatation and a hyperreflective rim (“bright rings”) outlining the upper part of the follicle. In healthy control subjects, bright rings, indicative of corneocyte accumulation in the follicular infundibulum during the early stages of comedogenesis, were undetectable(*p* < 0.05) [1]. In these early lesions, LC‐OCT revealed a non‐homogeneous, granular signal within the follicular lumen, likely corresponding to compacted keratinocytes or sebum, distinct from the large, organized plugs seen in advanced comedones. No perilesional inflammatory infiltrate was detected around microcomedones (*p* < 0.05), reinforcing that infundibular hyper keratinization is a primary pathogenic event rather than a consequence of inflammation [1, 15]. Indeed, our findings confirm hyper keratinization as the earliest event in acne pathogenesis [16], supporting the hypothesis of an “acneization field”, that is, the presence of abnormal follicles as observed with RCM, even before clinical lesions occur, in apparently healthy skin of acne patient [17].

In LC‐OCT vertical sections, closed comedones appeared as well‐circumscribed, ovoid cystic structures located immediately beneath an intact epidermal surface. The infundibular lumen was moderately enlarged compared to normal follicles, with a mean diameter of 300 µm at the skin surface level and 420 µm in large closed comedones. The luminal content was highly reflective and amorphous, consistent with dense keratin and sebum. In addition, most closed comedones showed a bright contiguous rim encircling the lumen, interpreted as a hyperkeratotic infundibular border, present either as a thin bright line or a thicker band if the cornified material was adherent to the follicular wall. Those findings closely match the histological morphology as retention cysts.

In contrast, open comedones appeared as markedly dilated follicular openings at the skin surface, with a funnel‐shaped structure. The lumen of open comedones was filled with abundant amorphous material characterized by a low reflectivity in the upper part of comedones, possibly corresponding to the compact melanin‐rich cap of the comedones, and a bright, granular material in the deeper portions, corresponding to densely packed keratin and sebum. Notably, open comedones contained multiple tiny bright particles or foci scattered throughout the debris, likely representing microcalcifications, trapped inflammatory cells, or hair fragments and hardened sebum.

Pathological progression involves a transition from simple hyperkeratosis, the initiating event manifesting as non‐inflammatory comedones, to inflammatory lesions, including papules, pustules, and nodules.

Papules presented as ill‐defined, solid nodules centered on a pilosebaceous unit, in contrast to the sharply demarcated cystic outline of a comedo. The overlying epidermis was often uneven or mildly acanthotic, partially effaced, while the underlying follicular lumen was replaced by a heterogeneous zone of reflectivity. Numerous minute ‘bright dots’ (5–20 µm) appeared both within the follicular epithelium (evidence of exocytosis) and spilling into the surrounding dermis. The “bright dots” may correspond to clusters of neutrophils and lymphocytes, dispersed throughout the papules. Radiating outward from the follicular core, a cloudy, hyperreflective halo extended roughly 100–200 µm into the dermis, corresponding to perifollicular inflammatory infiltrate.

Although pustules share many characteristics with papules, they are distinguished by a prominent, fluid‐filled cavity [18]. In vertical sections, this cavity appears as a round or oval, low‐reflectivity region filled with numerous bright dots, reflecting exudative fluid laden with dense inflammatory cells. The hyporeflective central was surrounded by a hyper‐reflective rim of leukocytes and fibrin forming a pseudo‐membrane.

The average cavity diameter was approximately 290 µm (range 200–400 µm). Typically located in the superficial portion of the lesion, the fluid‐filled cavity is located either within the epidermis (subcorneal pustule) or just beneath it at the follicular opening.

Our findings showed that LC‐OCT can visualize acne features from the earliest microcomedonal changes to fully developed inflammatory lesions, with high resolution. By detecting subclinical microcomedones, LC‐OCT can identify “acne‐prone skin” at the earliest stages of comedogenesis. Early recognition of these incipient follicular changes could enable personalized and tailored intervention, for example guiding early treatment with topical retinoids in patients with ongoing microcomedogenesis. However, the sample size was relatively small (52 lesions), our findings were largely descriptive, without histopathological correlation, and limited to 500 µm depth.

## 5 Conclusion

In conclusion, LC‐OCT could provide in vivo, rapid, non‐invasive, high‐resolution insight into the pathophysiology of acne. In an era in which advanced imaging modalities continually open new horizons into dermatological diagnostics [6, 19, 20], LC‐OCT may potentially become an innovative tool in acne research and management [21]. Further studies with larger cohorts and histopathological correlation are needed to confirm these findings and explore the potential role of LC‐OCT in clinical practice.

## Funding

The authors have nothing to report.

## Ethics Statement

All patients in this manuscript have given written informed consent for participation in the study and the use of their de‐identified, anonymized, aggregated data and their case details (including photographs) for publication. The study was conducted in accordance with the Declaration of Helsinki and approved by the local ethics committee.

## Conflicts of Interest

K. Peris has received consulting fees and honoraria from Abbvie, Almirall, Biogen, Celgene, Janssen, Galderma, Novartis, Lilly, Pierre Fabre, Philogen, Sandoz, Sanofi and Sun Pharma outside of the submitted work. D.O. Traini, G. Palmisano, A. Di Stefani, E. Bocchino, C. Guerriero and M.Suppa declare that they have no conflicts of interest relevant to this manuscript.

## Data Availability

The data that support the findings of this study are available from the corresponding author upon reasonable request.
